# Microbe-assisted crop improvement: a sustainable weapon to restore holobiont functionality and resilience

**DOI:** 10.1093/hr/uhac160

**Published:** 2022-07-22

**Authors:** Sandrini Marco, Moffa Loredana, Velasco Riccardo, Balestrini Raffaella, Chitarra Walter, Nerva Luca

**Affiliations:** Research Centre for Viticulture and Enology, Council for Agricultural Research and Economics (CREA-VE), Via XXVIII Aprile 26, 31015 Conegliano (TV), Italy; Department of Agricultural, Food, Environmental and Animal Sciences, University of Udine, Via delle Scienze 206, 33100 Udine (UD), Italy; Research Centre for Viticulture and Enology, Council for Agricultural Research and Economics (CREA-VE), Via XXVIII Aprile 26, 31015 Conegliano (TV), Italy; Department of Agricultural, Food, Environmental and Animal Sciences, University of Udine, Via delle Scienze 206, 33100 Udine (UD), Italy; Research Centre for Viticulture and Enology, Council for Agricultural Research and Economics (CREA-VE), Via XXVIII Aprile 26, 31015 Conegliano (TV), Italy; National Research Council of Italy - Institute for Sustainable Plant Protection (IPSP-CNR), Strada delle Cacce, 73, 10135 Torino (TO), Italy; Research Centre for Viticulture and Enology, Council for Agricultural Research and Economics (CREA-VE), Via XXVIII Aprile 26, 31015 Conegliano (TV), Italy; National Research Council of Italy - Institute for Sustainable Plant Protection (IPSP-CNR), Strada delle Cacce, 73, 10135 Torino (TO), Italy; Research Centre for Viticulture and Enology, Council for Agricultural Research and Economics (CREA-VE), Via XXVIII Aprile 26, 31015 Conegliano (TV), Italy; National Research Council of Italy - Institute for Sustainable Plant Protection (IPSP-CNR), Strada delle Cacce, 73, 10135 Torino (TO), Italy

## Abstract

In the past years, breeding programs have been mainly addressed on pushing the commercial features, forgetting important traits, such as those related to environmental stress resilience, that are instead present in wild relatives. Among the traits neglected by breeding processes, the ability to recruit beneficial microorganisms that recently is receiving a growing attention due to its potentiality. In this context, this review will provide a spotlight on critical issues of the anthropocentric point of view that, until now, has characterized the selection of elite plant genotypes. Its effects on the plant-microbiome interactions, and the possibility to develop novel strategies mediated by the exploitation of beneficial root-microbe interactions, will be discussed. More sustainable microbial-assisted strategies might in fact foster the green revolution and the achievement of a more sustainable agriculture in a climatic change scenario.

## Introduction

Agriculture and climate change are closely linked, as the agricultural sector generates significant amounts of gas emissions that strongly influence the climate and, in turn, augment frequency and duration of stresses [1]. The relevant increase of greenhouse gases in the atmosphere, the small but constant increase in temperatures, and the changes in the precipitation regimes strongly affect the quality and stability of agricultural production [2]. In this scenario, people need to protect crops from increasing environmental stresses by limiting the use of chemicals and adopting sustainable approaches as promoted by various national and international regulations and programs (*e.g.* Paris Agreement on Climate Change, European Green Deal, etc.) [3]. In the past years, breeding programs have been focused mainly on promoting commercial traits, neglecting several other important ones, such as those related to environmental stress resilience, which are indeed present in wild relatives [4]. Among these neglected traits, the ability to recruit beneficial, and functional, microbiomes is receiving increasing attention for its potential. Plants in fact, together with their associated microorganisms, are now considered as unique biological entity called holobiont.

To ensure their functionality, plants deploy their resources differently depending on environmental stimuli. Elite varieties, derived by long breeding programs, usually results into an unbalanced use of their resources, generally prioritizing growth and limiting the defence responses. The altered allocation of carbon resources (*i.e.* growth-defence trade-off) is one of the main features that can be restored by specific plant-associated microbiomes as recently demonstrated by few reports [5–7]. This negative trend is called “domestication syndrome” and it has been reported where intensively domesticated plants have lost their ability to survive on their own, away from the care of humans [8]. The domestication process has led to low self-support production systems with an enhanced need for external inputs such as substantial fertilization plans and a large use of pesticides [9]. Additionally, this process caused a dramatic reduction of plant genetic diversity leading to a significant impact of pathogens and pests on plant productivity and consequently an excessive use of chemical inputs to avoid excessive losses. Thus, the main side effect of plant domestication could be summarize as the loss of human neglected traits which are very important for wild plants fitness and their survival in natural environments [10], where plants live in association with thousands diverse microorganisms, with diverse outcomes depending on the interactions. Nowadays, most of the published articles about the agricultural application of microorganisms are focused on soil beneficial bacteria [11] or fungi [12]. Although many species of soil-bacteria or fungi capable of supporting plants have been identified, the next paragraphs will be focused on the plants interaction with two groups of microorganisms that are receiving growing attention for their potential in stressed environments: the mutualistic symbiosis formed by arbuscular mycorrhizal fungi (AMF) with the roots of almost all the terrestrial plants, including several crops, and the associations with Actinomycetes. By now, AMF are known to be one of the most important plant’s allies in the interaction with the surrounding environment, providing several ecosystem services to agricultural systems [13, 14]. Considering that plant roots in natural ecosystems are commonly colonized by AMF, the rhizosphere concept has been expanded to include the fungal component of the symbiosis, resulting in the term “mycorrhizosphere” [15]. The rhizosphere constitutes the microhabitat where fungal-bacterial interactions occur, with the fungi that affect the associated bacteria and *vice versa* (*e.g.* providing water and nutrients supply) [5]. However, there is a need to further strengthen the research to explore their potential to improve plant productivity and to restore the plant-microbiome equilibrium in agricultural system. For this reason, unearthing the mechanisms on which this fundamental cooperation is based on and trying to improve it by a more holistic view of breeding programs could be very promising. Additionally, domestication process has often modified the capacity of plants to interact with these fundamental soil microorganisms compared to the relative wild types [16–18]. Actinomycetes have been also shown to be very often part of the plant’s core microbiome [19–21]. They can be considered among the protagonists in the hidden world of plant-microbiome interactions. The application of next-generation sequencing (NGS) approaches to study microbial communities allowed to find the Actinobacteria phylum as one of the five most dominant bacterial phyla in soils [19–21].

Thanks to the peculiar capacity to live in wide range of temperatures and pH, and to change their morphology adapting to extreme environments, they are an ecologically divergent groups which is able to occupy a huge range of environmental niches [22, 23]. Furthermore, although Actinomycetes are important representatives of microorganisms beneficial for plants, their plant growth-promoting (PGP) traits, as well as their potential as biocontrol agents, have not been studied like for some other beneficial bacterial species such as *Bacillus* spp. and *Pseudomonas* spp. [24–26]. Several studies about Actinomycetes and their important role in supporting plants growth and wellness have been performed, but the dynamic interactions between them and plants are not still fully known, limiting the possibility to exploit these microorganisms in agriculture. Notably, it has been reported that these bacteria can enter in a close association also with AM fungi, giving an additional reason to contemporaneously analyze the potentiality of both Actinomycetes and AMF in improving plant performances [27]. In the last years, the application of biochar as amendment in agriculture has been also proposed and, in addition to an impact on carbon sequestration, a positive influence on rhizospheric beneficial microorganisms, including AM fungi, and microbial community network complexity has been reported in diverse plant species [28, 29].

Accordingly, this review will focus on examining in depth the critical issues related to the possibility to develop novel microbial-assisted selection of plants, optimizing rhizosphere/root-microbiome beneficial relationships, with a particular emphasis on AMF and Actinomycetes.

## Significant flaws of plant breeding: From domestication to new plant breeding techniques

As previously cited one of the most important traits that has been shelved is the ability of plants to interact with the thousands of microorganisms surrounding and supporting them in dealing with both biotic and abiotic stresses [30–34]. The domestication of plant populations is a co-evolutionary process, in which human selection of cultivated plant populations brings over changes in allele frequencies within these populations, making them more useful to human purposes and better adapted to the human-induced changes to the agro-environment [35]. It is now fundamental to underline how human-focused breeding has shaped plant traits involved in the interactions with microbiomes and how, in turn, the loss of these plant traits may negative influence the ability of current genotype to dealing with the surrounding environment. It is evident that anthropocentric breeding has profoundly altered the interactions between plants, insects, and their natural enemies. For instance, it has been reported that domestication process led to lower levels of volatile emissions during pest attacks as compared to wild relatives, thus affecting the attraction of natural enemies of pests and pathogens [8].

Production of resistant varieties through breeding programs is often slowed by the necessity to move polygenic resistances which requires several crossing cycles that, in case of woody plants, are laborious and time-consuming [35]. To overrun such issue, the common choice of breeders is to work with monogenic resistances which are easily manageable but likewise overcome by pathogens in short time [36, 37]. For example, the major resistance gene *Ty-1* was introduced to control tomato (yellow) leaf curl disease (TYLCV). However, plants shown differential responses to TYLCV strains [38] suggesting the importance of pyramiding multiple resistance genes improving the spectrum and resistance durability. This scenario was observed also in rice, indeed Qu and colleagues reported a time retained resistance level due to a rapid evolution of *M. grisea* [39]. Breeding programs for woody plants encounter additional limitations such as the high heterozygosity of elite cultivars, long juvenile stages that slow down the backcross steps and the movement of unwanted genes in the progeny that are linked to the gene(s) or QTL of interest (*i.e.* linkage drag) [40–42]. The latter aspect is often linked to modification of important commercial features, such as aromas, that generate new tastes and organoleptic profiles [43] that have to be acknowledged by consumers. For example, grapevine is characterized by different cultivars that are associated to the production of many aromas and therefore different commercial wines. The products of grapevine breeding programs lead to the production of new individuals with different characteristics from the parental cultivars that need to be registered as new varieties (with new commercial names) and in consequence the necessity of market acceptance [44, 45]. Thus, breeding programs on woody species result in laborious and time-consuming processes which can be quite easily overcame by pathogens and that in parallel negatively affect qualitative traits of fruits.

The possibility to exploit genome editing and cis-genesis technologies, based on the precise modification of DNA sequences, introduce a new shortcut for improving elite crop varieties [46–48]. The development and application of microbial-based products could be in more sustainable than classical and new genetic approaches, overcoming their limits, especially considering woody plants for which breeding programs are particularly slow and time consuming. Despite the interesting features of the new biotechnology approaches, diverse main problems should be in fact to be solved yet: i) strict regulatory rules are still diffuse [49], limiting the use of genome edited or cis-genesis varieties and ii) the ability in recruiting beneficial microbial consortia is not easily tackle through them. Additionally, these new genetic technologies may produce deleterious effects in crops by genome-wide off-target mutations, making the generation of novel tolerant/resilient crops by using them a little bit more complex than expected [50]. Beneficial microorganisms constitute an important target to enhance plant features, such as productivity and/or tolerance and resilience to environmental stresses thus reducing chemical inputs [51–56].

## Root traits to improve microbe-mediated climate resilience

A holobiont-level breeding strategy, in which microbes are one of the direct targets of the selection process, can originate a range of new phenotypes without changing plant genomic information [57]. Particularly, it could be very useful unearthing the ability of crops to assemble useful and healthy microbial communities. Several studies have already shown that diverse plant species are able to recruit specific microorganisms, establishing active interkingdom interactions that could be perceived as a “cross talk” [58, 59]. The “cry out for help” concept has been recently exposed in literature [60], considering root exudation as an adaptive mechanism by which stressed plants assemble health-promoting soil microbiomes [61, 62]. It has been demonstrated that plants can recruit beneficial bacteria upon pathogen infections, mainly disease resistance-inducing and growth-promoting ones [63]. The selection of plant phenotypes that efficiently interact and recruit taxa suppressing pathogens may alleviate the need to introduce disease resistances into the plant genomes [64]. Another important concept within the hidden world of holobiont interactions is the “soil memory”: from one plant generation to another, a given soil would hold its associated microbiota and thus, the wellness of plants can be improved, taking advantage of the pre-existing beneficial microbes for their development [65]. This plant/soil feedback is strictly due to the microbial legacies, which plants leave in soil, and it could be applied to the time scales necessary for the renewal of a olive grove, a vineyard, or at least an orchard, and could be exploited to restore the soil’s microbial functionality. Hannula et al. [66] have looked at the persistence and the impact of these legacies following a subsequent colonization by the same or different genotypes using six typical grassland plants [66]. The authors observed that microbial soil legacies, at the time of plant establishment, have a pivotal function in plant growth, concluding that soil microbiome legacies, although reversible and versatile, can create plant/soil feedbacks through the alteration of the endophytic communities developed in the course of early ontogeny. Additionally, the host genome is highly conserved with slow genetic changes, especially in perennial plants. Genomes of microbiomes, instead, are dynamic (*e.g.* horizontal gene transfer, mutation) and can change rapidly by modifying microbial populations in response to environmental changes [67]. Considering the climate change scenario and the consequent increase in the incidence of both abiotic (*e.g.* drought and salinity) and biotic stresses (invasive pathogens), holobionts with functional and dynamic microbiomes can better adapt to the occurrence of different stresses when necessary. A full understanding of the mechanisms governing the selection of microbial communities by the plants will enhance the development of new strategies to improve the agriculture future.

**Figure 1:**
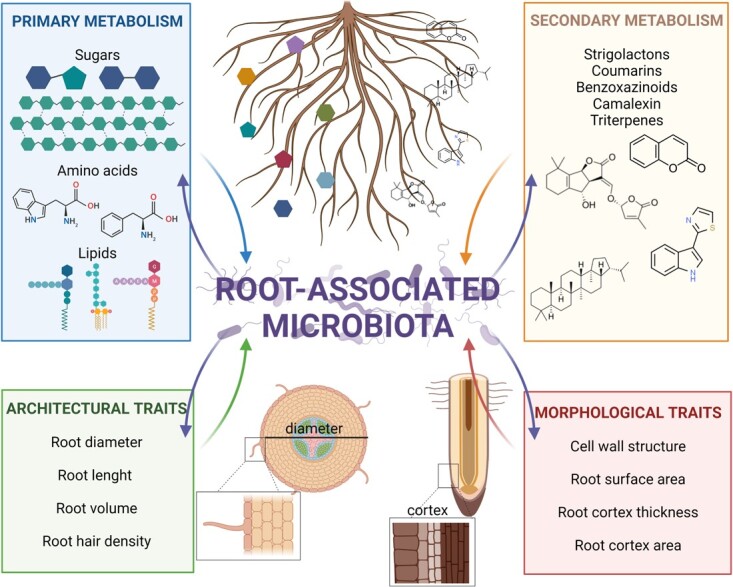
**Representation of the reciprocal influence that root-associated microbiota and plant features exerts each other.** In the upper part, metabolic features usually associated to root exudates that interact with root-associated microbiota. In details, both molecules from the primary metabolism (*e.g.* sugars, amino acids, organic acids, etc.) and from the secondary metabolism (*e.g.* strigolactones, flavonoids, terpenoids, etc.) are involved in the recruitment of beneficial microbes as well as in shaping the soil microbiota. In the lower part, root architectural and morphological traits that influence the interaction with soil microbes and shape the composition of the root-recruited microbiota.

Since plants are sessile, they need to mine soil for finding important resources such as water and nutrients (*e.g.* phosphorous, nitrogen, potassium, etc.), whose distribution is patchy and change rapidly over time. Modulation of the root system architecture, root anatomy and chemistry are the plant responses to this challenging environment, allowing them to explore soil, detect and exploit nutrients and water [68]. Additionally, plants can also shape the root-associated microbiomes to improve their foraging activities. Reciprocally, soil microbes can trigger important adjustments in root development, physiology and chemistry [69] (Figure 1), creating a dynamic interplay that impact on plant nutrition and health modulating the growth-defence trade-off [70]. It is evident that, over the time, breeding programs aimed at improving productive features, often not considering the root traits as an important aspect to be characterized and associated to beneficial and functional microbiome structures [71].

Remarkably, root architecture as well as morphology are deeply involved in resource acquisition, and breeding for different root ideotypes have been suggested as promising targets for climate resilient crops also thanks to an improved rhizosphere microbiome [72]. Root architecture encompasses the spatial configuration of the whole root system including pivotal traits such as root length, density, branching, angle, and total biomass [73]. Root morphology, on the other hand, encompasses physical traits of each single root, such as cell wall structure, root hairs, diameter and surface area [73]. Interestingly, hints of reciprocal relationship between soil microbes and root architecture/morphology were already reported. Inoculation with single-strains highlighted the ability of specific rhizosphere bacteria to modify root architecture and morphology through the production and the release of key plant phytohormones (*e.g.* auxins and cytokinins) [74]. Furthermore, recent reports demonstrated an increase of root length, volume and branching in wheat and soybean when inoculated with specific microbial isolates [75–77]. Despite these interesting reports on root architecture, effects of microbial inoculations on root morphological traits are less clear, as demonstrated by the inoculation experiments in rice, wheat, or soybean, where the same isolates displayed increased, decreased, or no effects on root diameter respectively [76, 78, 79]. Thus, sustainable agriculture through inoculation of microbial consortia is a feasible route, but it still remains a gap that have to be filled by studying root traits and the effects on soil and root-associated microbiomes. This knowledge will result in pivotal information exploitable for breeding program aimed to restore the precious ecological services offered by beneficial microbiomes.

In addition to architecture and morphology, root exudates, both from primary (particularly sugars, amino acids, and organic acids) as well as secondary metabolism (*e.g.* flavonoids and strigolactones), play key roles in defining symbiotic relationships [80] and, consequently, changes in exudates composition might limit or negatively influence these positive interactions. Considering survival of root-associated microbial communities, plants can support the proliferation of soil microbiota releasing carbon substrates through the root system [81]. Different studies also highlighted that an high plant diversity was associated with high microbial diversity [82, 83], confirming that when exudate mix from several plants were added to monocultures, an increase in microbial diversity was observed [84].

**Figure 2:**
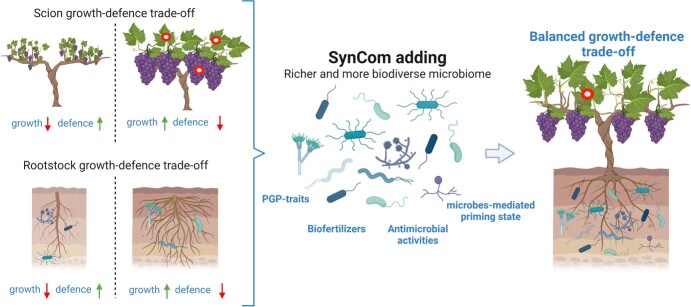
**Schematic representation of SynCom adding traits for balancing the growth-defence trade-off in grafted crops.** Depicting different (culturable) microbial populations, associated to diverse environments, can allow the development of SynCom that can in turn modulate the growth-defence trade-off, leading to more resilient plants showing balanced growth-defence features.

In this respect, it is worth noting to consider the potential ecosystem services conferred by root systems of cover crops in the agro-ecosystems. Cover crops species are often ignored since most of them do not provide a direct economic income to growers underestimating their positive roles for belowground features (*e.g.* resources capture, improvement of microbial biodiversity and soil physico-chemical characteristics [85]). Although researches investigating the relationships between cover cropping and soil microbiomes are still limited, promising results in terms of positive effects on soil microbial abundance, diversity and functionality have been reported in many agricultural systems [86]. Furthermore, emerging studies outlined the ecosystem services provided by roots of diverse cover crops such as: i) improved soil structure and stability thus limiting water runoff and topsoil displacing (*e.g.* roots of grasses prevented soil erosion [87] while tap-rooted plants as forages, alfalfa or chicory can easily penetrate compacted soil layers favoring soil aeration and water infiltration [88, 89]); ii) enhanced soil resource capturing, improving soil nutrition and fertilizers use efficiency (*e.g.* increase of soil nutrients by tall fescue or chicory [89]; nitrogen increase by leguminous cover crops such as pea, vetch or alfalfa thus reducing chemical fertilizers needs [90]); iii) improved soil microbiome biodiversity and organic matter content mediated by root exudates of which composition greatly varied among cover crops species, positively influencing soil microbiome structure and functionality [91]. Taken together these findings highlight the importance to enhance cover crop root traits selecting those able to enhance ecosystem services in the agricultural contexts as (near) future challenge for breeders. Even if increasingly attention were recently posed by several scientists, to date cover crops have been subjected only to minimal domestication and breeding selection with respect to cultivated crops [92]. However, in the frame of an holistic view about microbe-assisted improvement of crop (and agro-ecosystems) resilience, breeding programs, or the use of novel genetic tools able to exploit superior root traits of specific cover crops (*e.g.* targets to improve rooting depth or root exudates to increase beneficial microbes recruitment/biodiversity), will be able to provide high impact to the environment and farmers at low cost.

Further efforts are needed to develop novel breeding approaches more focused to protect and improve the interactions between plants and the associated microbial communities, also restoring growth-defence trade-off balance in host plants (Figure 2).

## Customized genotype- and environmental-specific SynComs to boost plant resilience

The use of multi-omics approaches (*i.e.* so called holo-omics [93]) to study the functionality of plant-microbiome ecosystems led to the generation of data on multiple levels [92], focusing on diverse targets (DNA, RNA, protein or metabolites), as well as to the characterization the plant associated microbiota [62, 94]. These data are particularly relevant, resulting in obtaining information on the ability of specific genotypes to recruit specialized microbial strain(s) or consortia and providing information that might be useful in the manipulation of these interactions. The development of next-generation DNA sequencing platforms, and the integration of data from diverse omics approaches, have facilitated the exploration of the complexity of the plant-associated microbial communities in a wide range of environments. Corbin et al. [95] proposed a framework to identify genes involved in plant-microbe interactions *via* stochastic perturbation of DNA methylation patterns. Exogenously induced DNA demethylation can randomly generate new epialleles in a plant population, that can subsequently alter gene expression of genes and thus the plant phenotype (including the associated microbiomes). The combination between individual changes in DNA methylation (novel epialleles) and phenotype (novel microbial community composition and functions) can be determined using epigenome wide association studies (EWAS) and plant gene expression analysis followed by the evaluation of metabolites production as a validation step. Interestingly, Huang et al. [96] showed how *Arabidopsis thaliana* can assembly and shape its root-associated microbial community producing a variety of specialized triterpenes [96]. Bulgarelli et al. [97] suggested a prevailing recruitment model for root-microbiota assembly based on the relative abundance of specific microbial taxa. Mainly basing on 16S rRNA sequencing, the relative abundance of bacterial taxa in soil suggested that bacterial root community forms by two-step or multiple-step selection process, being dense in bulk soil and becoming more differentiated and enriched for specific phyla from rhizosphere to root. In contrast, Wang et al. [98] suggested a novel amplification-selection model useful to quantify rhizosphere microbiota assembly, sustaining that the relative abundance of microbial 16S rRNA gene sequences does not correctly reflect the absolute abundance of bacteria. The microbial communities were quantified in bulk soil, rhizosphere and roots of two different plants (*Medicago truncatula* and *Oryza sativa*), showing all the dominant bacterial phyla more abundant in the rhizosphere than in bulk soil, and an additional host specific selection of bacterial phyla in roots. The augmentation of diverse phyla in the rhizosphere reflected an increase in nutrient availability in this compartment, while the lacks of some bacterial taxa might depend on several factors such as nutrient availability, growth rate and the interactions with other microorganisms. Looking at the tolerance and resilience to abiotic stresses, Zolti et al. [99] described the taxonomic variations and the functional responses upon long-term irrigation with water differing for its quality (fresh water *vs* treated wastewater).

As previously mentioned, an interesting aspect uncovered by the multi-omics approach is the so called “soil memory”, namely the opportunity to alter the soil microbial communities planting specific plant species [100]. Thanks to computational approaches it is possible to verify the most important microbial taxa that can influence the composition of a specific environment-associated microbiome, that can be identified as the soil core microbiota [65]. In this line, it is possible defining the impact that specific taxa have on the recruiting of others that in turn influence diverse plant functions [101].

**Figure 3:**
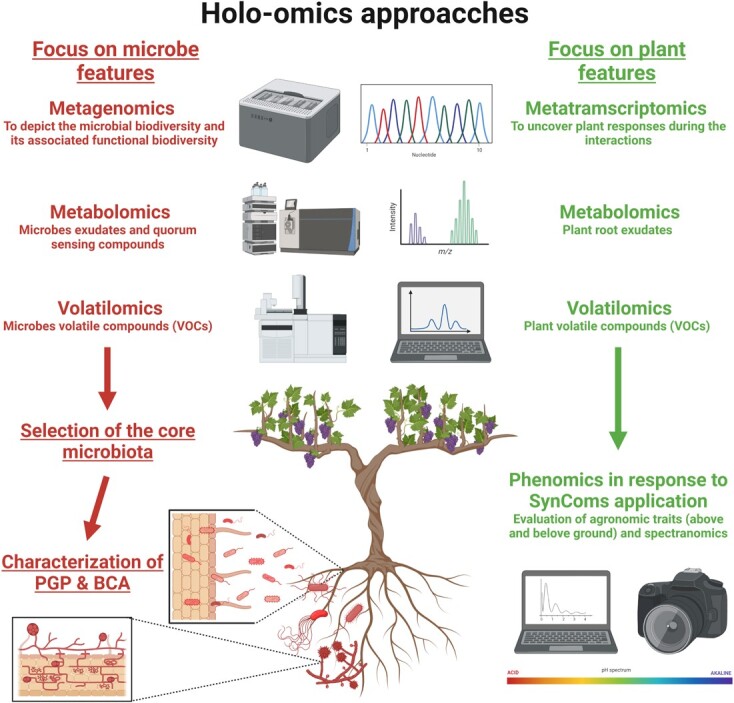
**Workflow for the characterization of customed SynComs.** The same -omics approaches can be exploited for the characterization of both plant and microbe features. From the top, sequencing techniques can be used to characterize the microbial profile and/or specific isolates (*e.g.* microbiome profiling, microbial genome sequencing, etc…) as well as plant responses under specific conditions. Then, metabolomics and volatilomics approaches can be used to identify key metabolites involved in the interactions between microbes and its host. Once the core microbiota has been selected, the characterization of plant growth-promoting features and biological control potential will be evaluated. Finally, phenomics approaches (applied both on root and canopy) can be exploited to detect plant responses when exposed to the developed core microbiome.

The SynCom approach can be defined as an interesting laboratory approach to study plant-microbes interaction excluding other environmental effects, limiting the complexity of the experimental system [102]. To formulate a valuable SynCom it is necessary to collect several information from the holo-omics and from the microbe behavioural side. In details, the formulation of a core microbiota [101] is grounded on the identification of keystone species: a group of well described microbes with known PGP-traits (see next paragraph) and antagonistic activities that have no human or animal pathogen features [103]. Zhuang and colleagues [104], have adopted high-efficiency top-down approaches based on high-throughput technology and synthetic community approaches to find plant-growth promoting bacteria (PGPB) in garlic rhizosphere. They have found out that bacteria belonging to the *Pseudomonas* genus were key PGPB in the rhizosphere of garlic and, subsequently, SynCom with six *Pseudomonas* strains isolated from the garlic rhizosphere was assembled, showing the ability to promote plant growth. Such microorganisms are fundamental since they have naturally evolved in close cooperation with a specific plant’s genotype and phenotype and so they can be considered in a breeding program grounded on the improvement of the holobiont [105]. Furthermore, Paredes et al. [106] have recently developed a new method, based on synthetic communities approach, −omics techniques (e.g. RNA-seq) and neural network (NN) prediction, to design and test bacterial communities altering the plant response to phosphate starvation [106]. As a first step, a bacterial collection has been classified (plant-bacterium binary-association assays) according to the effect on plant Pi content achieving the design of bacterial synthetic communities. Then, the *Arabidopsis* phenotypes with the synthetic communities have been evaluated (*e.g.* Pi content, roots elongation and plant transcriptional profile) and the prediction of Pi content for new hypothetical synthetic communities has been achieved by using NN. Finally, they validated the NN predictions evaluating the performances of *A. thaliana* with the new developed synthetic communities. Thus, this strategy allowed to design and test small consortia of bacteria with predictable host phenotypic outputs, discovering the best synthetic community for a specific host genotype. Nowadays the synthetic community approach has been exploited mainly as reductionist model to understand the plant microbiome assembly and the output obtained from field applications of experimental SynComs has been often contrasting, due to the fact that non-specific additive microbial cocktails are sub-optimal for general application [107, 108]. This can be related to genotype- and environmental-dependent effects. Additionally, only limited data on the mechanisms at the basis of the interactions with beneficial microorganisms in natural conditions are available, rendering still unstable their exploitation in agriculture [109, 110]. Nowadays, however, thanks to the emergence of next-generation sequencing, the application of complementary omic-tools, and considering the differences among their outputs, deep insights into the diversity and composition of the bacterial communities associated with diverse host, the characterization of plant-microbiome interactions and the selection of the best performing SynCom for a specific genotype and environment could be reached (Figure 3). Specific examples of SynCom formulations developed during the last 10 years for several purposes and their relative outcomes have been summarized in Supplementary Table S1.

**Figure 4:**
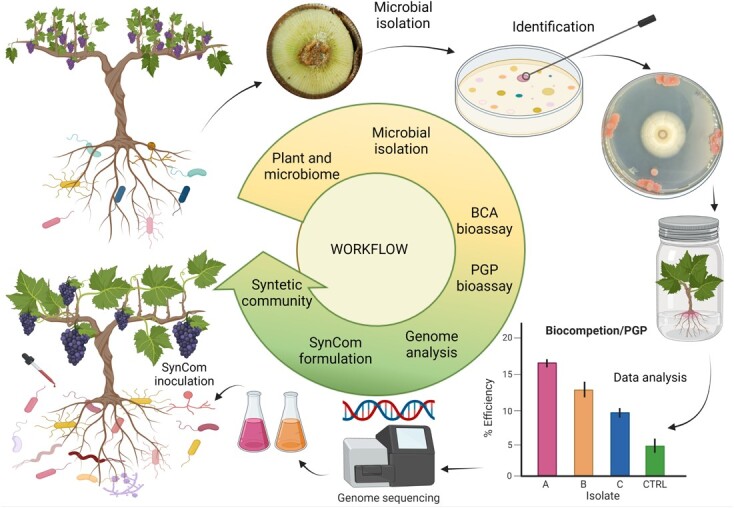
**Workflow for the development of a SynCom.** Starting from a specific environment and/or wild well-adapted plant population, the first step is to isolate and identify the culturable microbial endophytes. Thus, the identification and selection of potentially beneficial microbes occur through several *in-vitro* tests (*e.g.* biocompetition against phytopathogens and assessment of plant growth-promoting traits). Finally, prior to SynCom formulation, it is highly desirable to perform genomes sequencing of the best performing microbes (at least for bacteria) to have a clear picture of the biosynthetic pathways present in their genomes and to avoid the selection of isolates which can potentially produce metabolites with detrimental effects on animals and humans.

SynComs plant-customized with high-throughput methods (such as metagenomics and meta-transcriptomics) can address problems commonly faced with microbial field applications [111]. It is thus possible to screen beneficial microorganisms for predicting their establishment and functioning in different natural environments, defining a range of microbial functions associated to diverse strains in different conditions (under a gradient of pH, temperature, and water and nutrient concentration, etc.) [106]. Additionally, the compatibility between host plants and microorganisms may be evaluated in different pairwise combinations. Furthermore, this approach may offer a flexible and powerful tool suiting the needs of individual farmers. The same reference plant genotype could be combined with different microbiota to generate easily customized phenotypes [57]. Starting from modern phenomics approaches (high-throughput plant phenotyping), several traits related to growth, yield, and adaptation to stress can be precisely evaluated and the screening of eco-physiological and agronomical traits can be simultaneously performed [112]. From these collected data, the selection of the best performing phenotypes living in a specific environment can be easily viable. Once phenotypes selection has been done, it is possible to screen the related hologenome by complementing the data obtained from metagenomics and other omics techniques (*e.g.* metatracriptomics and metaproteomics), achieving thus a detailed hologenome picture leading to define the core beneficial microbiome strictly linked with the involved genotype growing in a particular environment [113]. After a customized SynCom development and application, the evaluation of different plant physiological parameters and the dissection of plant responses at molecular level (through RNA-seq, proteomics, metabolomics and volatilomics) are necessary to confirm the ability of these customized SynCom [114]. A crucial still open question is how to implement this strategy on an industrial scale. As a first point, industries have to keep records of microbial characteristics such as name and function, etc. Similarly, they should collect data on ecological features, *e.g.* survival in different types of physical environments, compatibility with crop variety, and mutual antagonism [115]. If these data will be available, then customizing personalized microbial consortia will be feasible [116]. Collected information could be also analysed using specific software (*e.g.* decision supporting system - DSS) that will further minimize the need of experts for such customizations [115]. In addition, looking to wild growing species, it is feasible to find microorganisms that can confer important plant traits lost during the domestication and/or breeding programs [117, 118]. After the identification and characterization of target microorganisms, building a personalized SynCom and inoculating it in agricultural systems might be a very promising tool to restore and improve beneficial microbial communities which have been previously damaged due to long time of anthropocentric breeding [119, 120] (Figure 4).

## Soil beneficial bacteria: Focus on Actinomycetes, promising allies to support holistic breeding programs

During the last few years, a great number of studies have been dedicated to searching out for soil beneficial plant-associated bacteria (Supplementary Table S2) [121], mainly focusing the interest on *Bacillus* and *Pseudomonas* species that have showed promising attitude to enhance plant growth and wellness. Diverse aspects of *Pseudomonas* and *Bacillus* spp. as elicitors of Induced Systemic Resistance (ISR) and as direct antagonists towards different pathogens have been largely described, suggesting that some strains might achieve significant reductions in the incidence/severity of diverse diseases on several plants [122, 123]. These species are characterized by several PGP traits, *i.e.* the production of phytohormones or siderophores, the solubilization of nutrients, *i.e.* phosphorus, and the capacity to fix atmospheric nitrogen [124, 125]. Moreover, both these genera have showed great potentiality in alleviating damages of abiotic stresses like extreme temperatures [126, 127], water stress [128] or high salinity [129]. As an example, *Bacillus cereus* KTMA4 has been reported to produce molecules involved in growth-promoting and tolerance such as Indole-3-acetic acid (IAA), ammonia, siderophore and 1-aminocyclopropane-1-carboxylate (ACC) deaminase. It has been demonstrated that tomato plants obtained from seeds treated with this strain showed an increasing in seed germination percentage and an inhibition against major tomato phytopathogens [130]. Considering *Pseudomonas* species, Noori et al. [131] showed the potentiality of *Pseudomonas fluorescens* in enhancing plant growth and in dealing with the dangerous cereals pathogen *Pyricularia oryzae*. Twenty *Pseudomonas* strains, isolated from the rhizosphere soils of paddy areas in Malaysia, were screened for their plant growth promoting activity, showing the ability for siderophores’ production. Additionally, fifteen strains were positive for IAA production and eighteen isolates for phosphate (Pi) solubilisation. All the twenty bacterial isolates also inhibited the pathogen *Pyricularia oryzae* in an *in vitro* experiment [131].

In addition to this well-studied bacteria, diverse species belonging to Actinobacteria phylum have shown to be very often a promising part of the plant’s core microbiome [19–21] and they can be thus considered as main actors in the hidden world of plant-microbiota interactions. The use of NGS technologies to describe the microbial communities allowed to verify that this bacterial group is one of the five most dominant bacterial group in soils [19–21]. Moreover, differently from other phyla, Actinomycetes present the capacity to live under the most diverse conditions such as aerobic and anaerobic environments as well as different temperatures and pH. Furthermore, they are involved in the catabolism of complex molecules (*e.g.* diverse plant cell wall components, proteins and lignin), achieving a nutritional advantage and giving them a high chance to survive and compete for the colonization of natural ecological niches [23, 132, 133]. These bacteria also present other exploitable features such as the production of a wide range of secondary metabolites of agricultural values [134–136] and are important for the plant health, forming associations with some non-leguminous plants and fixing atmospheric N (*Frankia* genus) that is then available to both the host and other nearly plants [137]. Overall, the results already present suggest that Actinomycetes have several properties that make them good candidates for the biotechnological exploitation in agriculture, mainly in light of the climate change already ongoing [138, 139]. Regarding the allocation of carbon sources in plants, Actinomycetes seem to be a promising tool to modulate the plant growth-defence trade-off since they are able to improve both the growth and the resilience of plants to stress conditions [140]. They can also be considered as biofertilizers thanks to their PGP-traits and so they may be considered for improving yield in genotypes characterized by low productivity [141, 142]. Kim et al. [143] highlighted the effects of two microbial inocula, one containing two *Methylobacterium oryzae* strains (CBMB20 and CBMB110) and one with the addition of three species of arbuscular mycorrhizal fungi (AMF), on the growth of red pepper (*Capsicum annum L*.). The use of *Methylobacterium oryzae* strains led to a significant increasing in root length and root fresh weight with respect to untreated control plants [143]. Additionally, the inoculation of *M. oryzae* strains and AMF significantly increased diverse growth parameters and chlorophyll content in comparison with uninoculated control plants [143].

Several studies have shown the Actinomycetes capacity to inhibit the growth of different pathogens, thus limiting disease incidence and severity [144, 145] (Supplementary Table S1). They can act through both a direct antagonism towards pathogens [144, 146] and by activating a state of priming in the plants [147]. In this respect, Zothanpuia et al. [148] used dual culture *in vitro* assay to screen twenty-two actinobacterial strains against diverse fungal, including diverse *Fusarium* species, and bacterial pathogens, such as *Staphylococcus aureus, Pseudomonas aeruginosa, Micrococcus luteus, Bacillus subtilis and Escherichia coli*, in addition to one yeast pathogen (*Candida albicans*), providing information on the most promising strains for antimicrobial activity against both bacterial and fungal pathogens.

An ability to enhance plant responses to face up some of the main abiotic stresses such as extreme temperature, drought and salinity was also reported [149] (Supplementary Table S2). Confirming the role of actinobacteria to increase drought tolerance in plants, Yandigeri et al. [150] demonstrated that the co-inoculation of three different endophytic actinobacteria (*Streptomyces coelicolor* DE07, *Streptomyces olivaceus* DE10 and *Streptomyces geysiriensis* DE27) will lead to significant enhancement of seedling features, growth and yield in wheat upon water limitation. Additionally, actinobacteria seems to have a potential role being able to live in environment characterized by extreme temperature. Kurapova et al. [151] conducted a study in Mongolian desert soils observing that actinomycetes strains with thermotolerant and thermophilic characteristics were present in abundance and, among the thermotolerant individuals, members of the order *Actinomycetales, Streptomyces, Micromonospora, Actinomadura,* and *Streptosporangium* genera.

However, although many works about the efficiency of *in vivo* applications of Actinomycetes bio-inoculants, both as biofertilizers and biocontrol agents, have been already performed [152, 153], their potentiality has not yet been adequately explored. Thus, it would be very useful unearthing the full potential of this bacterial phylum and implementing their application in agricultural environments, also considering the possibility to formulate them in SynComs (see previous paragraph).

## Focus on arbuscular mycorrhizal symbiosis responsiveness as a trait for breeding

Among beneficial root-associated microorganisms, AMF are considered the most important bio-fertilizers. These microorganisms, as symbiotic fungi, colonize plant roots of several crop species and help the host plants in the uptake of water and nutrients, by receiving in turn carbohydrates and lipids compounds [154, 155]. Additionally, these molecules are thought to be exported out of the root cells across the periarbuscular membrane to be exploited by the fungus [156]. Besides an enhanced nutrition, mainly related to an improvement in phosphate (Pi) uptake that particularly occur in limiting nutrient conditions, several papers have described the impact of AM fungi on plant tolerance under abiotic stresses such as drought, salinity, and cold conditions [157]. Since AM associations are broadly present in cultivated soil from diverse environments and they form symbiosis with the roots of major crop species, their potential to improve crop productivity is an opportunity for plant breeding that should be more exploited. In addition, it is worth noting that developing crops with higher P-use efficiency is an important goal for breeders [158]. Recently, it has been suggested that both direct and indirect pathways (this last *via* AM symbiosis) that most plant species utilize to ensure phosphate are regulated by the same phosphate sensing-centered pathway. These findings, leading to the recognition of several actors of the phosphate starvation response-centered regulatory network involved in AM symbiosis, might be useful to assist breeding in the generation of plants that use P more efficiently [159]. However, this goal is generally addressed mainly focusing root traits in diverse genotypes without considering the interactions with soil microorganisms. As recently suggested [160], breeding approaches to improve the results from beneficial plant-fungus interactions should be obtained through the selection of traits of both symbionts (*i.e.* the plant and the fungus) involved in the association establishment and functioning. It will be important that future breeding strategies takes in account the interaction of root traits with symbiosis-related ones, with the aim to achieve optimal production also reducing application of fertilizers (mainly P-based products). An increasing number of studies report that AM responsiveness varies among plant accessions [161, 162]. An important point that should be developed is related to the characterization of additional host genotypes, including landrace and wild-relative whose diversity should be more explored [163]. The evaluation of mycorrhizal dependency in diverse plant species accessions has been performed since a long time. A comparison among varieties of wheat generated before and after 1990 suggested that the oldest varieties were more responsive to AM colonization than those obtained later [164]. Thus, plant breeding under high nutrient conditions has selected wheat lines with an increased phosphorous demand contrary to the capacity to form AM interactions. However, the impact of breeding on symbiosis effectiveness is still under debate [165], it is incontrovertible that plant breeding inadvertently selected for a reduction in dependence on AM symbiosis and not for a loss of compatibility, leading to modern cultivars with reduced but still retained ability to form AM symbioses. Genotype-dependent plant responses to AMF colonization have been demonstrated on biomass, yield and physiological features, while less is known when it comes to those for AMF-mediated disease resistance [166]. It has been proposed to include disease resistance as a trait for mycorrhizal responsiveness and it is worth noting that, to observe differences in the efficiency, genotype selection needs to occur in environments that do not suppress the plant–microbe interaction [166]. As for the classical breeding, novel breeding protocols evaluating a genotype responsiveness to AMF colonization could takes advantage from the development of protocols for the high-throughput phenotyping platforms, allowing to test many plants contemporaneously. The combination with high-throughput genotyping systems already led to the identification of quantitative trait loci (QTLs) linked to host benefit, supporting the feasibility of breeding crops to maximize profit from symbiosis with AMF [167]. In addition, QTLs with a role in colonization have been reported in several crops [167, 168]. A relevant bottleneck that should be considered in field studies is the lack of appropriate AMF free controls when an exogenous AM fungal inoculum is applied to soil, rendering difficult the evaluation of the efficiency of the AM symbiosis in agriculture. Although in the last twenty years great advancement have been done in the ecology and biology of these interactions, most of the experiments were carried out in greenhouses or growth chambers, while only limited studies have been conducted in open-field conditions [169]. Additionally, some plant and AMF combinations are more productive than others, and the nutrient status of soils also affects the species composition of AMF and the success for the symbiotic interaction, complicating the real application of these beneficial microorganisms [6]. In parallel to breeding protocols that consider the potential to form AM symbiosis as a priority trait, a successful strategy could be to maintain and improve the soil AMF potential with the use of soil managements with a low-impact on soil microbial communities [170]. Interestingly, the *Rhizophagus irregularis* non-symbiotic growth and spore production were reported in the presence of an external supply of certain fatty acids, *i.e.* myristates [171]. In this line a useful application for agriculture could be the developing of crop plants for myristate production with the aim to have AM fungi-friendly crops [172]. Additionally, the application of myristates could enhance the AM fungal biomass *in loco*, leading to a reduction in an external inoculation. This should be particularly relevant, and directly applicable in agriculture, often where AM fungal abundance is suppressed by a range of invasive agricultural management practices [170].

## Conclusion

The ongoing climate change is seriously threatening the food access for billions of people and the neglect of environmental or ecosystems health and associated loss of biodiversity is a critical issue further worsening the health of the agricultural systems. Thus, the challenge of maintaining adequate yield and quality of food and feed under unrelenting climate changes is formidable. Improving or developing new eco-friendly management strategies, able to restore part of the loss biodiversity, and selecting stress-adapted genotypes represent sustainable approaches that are now under scrutiny. Notwithstanding, an essential step to face this challenge should be to take into account the roles played by root-associated microbes and exploit the hidden potential that is starting to unearth. Development of SynComs adapted to specific agro-environmental conditions is the beginning of a regenerative path that will not consider the plant as a stand-alone entity but as a complex organism composed also by the associated microbiota (*i.e.* the holobiont). In the light of what discussed, exploiting the potential of microbes to improve wellness, resilience and product safety of crops seem to be a promising path to overcome the ongoing climate change and preserve food yield and quality for the future.

## Acknowledgement

Part of this work was funded by the PRIMA – REVINE project (Italian MUR DM n.1966/2021, Project ID 20114-2) and by the MicroBIO project (Funding ID: 2021.0072 - 51886) funded by Cariverona foundation.

## Conflict of interest

The authors declare no conflicts of interest.

## Supplementary data

Supplementary data is available at *Horticulture Research* online.

## Supplementary Material

Web_Material_uhac160
