# Supplementary material for: Microbe-assisted crop improvement: a sustainable weapon to restore holobiont functionality and resilience
Source: Hortic Res. 2022 Jul 22;9:uhac160. doi: 10.1093/hr/uhac160 (PMC9531342; doi:10.1093/hr/uhac160)
Supplement: Web_Material_uhac160 [file web_material_uhac160.docx]

**Table S1.** Examples of SynCom formulations developed and tested *in vivo* during the last 10 years.

| **Plant species and condition** | **SynCom source and formulations** | **Main outcomes** | **Reference** |
| --- | --- | --- | --- |
| Maize (three commercial hybrids grown under glasshouse conditions) | 17-strain community formed by bacteria isolated from Sugarcane root and stalk tissues | Upon severe drought SynCom in inoculated plants positively increase resilience to drought modulating sap flow and improving the water use efficiency as well as other real-time phenotyping parameters analyzed | Armanhi et al. (2021)^1^ |
| *A. thaliana* (plants grown in sterilized substrate) | Two SynComs formed by 218 leaf-derived bacteria and 188 root+soil-derived bacteria | Inoculation with leaf- or root+soil-derived SynComs showed that specific SynCom can colonize their respective organs displaying competitive advantages | Bai et al., (2015)^2^ |
| *A. thaliana* (55 mutant plants grown in axenic conditions) | 7-strain community representing the core microbiota composed by 4 Alphaproteobacteria, 2 Actinobacteria and 1 Betaproteobacteria | The authors concluded that SynCom composition is influenced by host genetic variation helping to identify novel host genes directly involved in phyllosphere microbiota structure and dynamics | Bodenhausen et al. (2014)^3^ |
| *A. thaliana* (gnotobiotic plants) | 62-strain community assembled composed by 32 Proteobacteria, 20 Actinobacteria, 6 Bacterioidetes, 4 Firmicutes | In this study the authors deciphered the principles determining the community development and its structure dynamics after inoculation. The early timing of microbiome manipulation is essential to obtain a stable *in planta* SynCom | Carlström et al. (2019)^4^ |
| *A. thaliana* (mutants with altered phosphate starvation responses. For SynCom experiments plants were grown in axenic conditions) | 35-strain community isolated from roots of *Arabidopsis* and Brassicaceae species | The inoculated SynCom positively influenced plant responses under phosphate-limiting conditions establishing a direct link between plant nutrition and expression of genes related to the immunity responses | Castrillo et al. (2017)^5^ |
| Sorghum (4 genotypes with contrasting N-use efficiency. Potted plants grown under glasshouse conditions) | Five SynComs formed by a total of 36-strain community bacteria isolated from sorghum roots and bulk soil grown in high- and low-Nitrogen field contents | Plants inoculated with the diverse SynComs showed growth features that are genotype- and plant N status-dependent. The same factors determined the colonization pattern by bacterial SynComs | Chai et al. (2021)^6^ |
| *A. thaliana* (gnotobiotic plants) | Seven SynComs formed by 148 bacteria, 34 fungi and 8 oomycetes mixed in diverse combination | SynComs were used to re-populate gnotobiotic plants at multi-kingdom level showing maximal plant growth and survival. These findings demonstrated that over the evolution plant as host favoured interkingdom microbial assemblage rather than with a single microbial group | Durán et al. (2018)^7^ |
| *A. thaliana* (plants grown in axenic conditions) | Two SynComs formed by 218 leaf-derived bacteria and 188 root+soil-derived bacteria | This study provides new insights about plant microbiome modulation by pathogen (powdery mildew) which affect host source-sink relationships and immune responses. SynComs applied did not altered the powdery mildew infection. | Durán et al. (2021)^8^ |
| *A. thaliana* (plants grown in axenic conditions under diverse P concentrations) | 3 SynComs formed by bacteria isolated from Brassicaceae roots | The authors dissected plant-microbe interactions under different P concentrations, linking phenotypic shifts with changes in microbiota composition | Finkel et al. (2019)^9^ |
| *A. thaliana* (plants grown in axenic conditions) | 185-strain community formed by bacteria isolated from Brassicaceae roots | The authors demonstrated that a single genus, *Variovorax*, is the key for maintaining root growth by manipulating host hormone balance | Finkel et al. (2020)^10^ |
| *A. thaliana* (plants grown in axenic conditions) | 14 partially overlapping SynComs | In this study the complex interactions among host phenotypes, microbiota and environment have been investigated. The presented approach defines the rational design and deployment of microbes to improve host performances | Herrera Paredes et al. (2018)^11^ |
| Tomato (plants grown in natural soil) | Community of 8-*Pseudomonas* strains with proved biocontrol activities against the bacterial pathogen *Ralstonia solanacearum* | *Pseudomonas* SynComs survival in natural soil is enhanced with increased *Pseudomonas* community diversity and, in turn, decreasing *R. solanacearum* disease incidence | Hu et al. (2016)^12^ |
| Maize (plants grown in pot with sterilized substrate under glasshouse conditions) | Two SynComs formulated using bacterial strains isolated from i) bulk soil (15-strain community) and ii) maize roots (12-strain community) | In this study the SynCom approach was used to understand bacterial colonization and assembly as well as plant-microbe interactions under organic (phthalate) pollution | Huang et al. (2022)^13^ |
| *A. thaliana* (mutants plants defecting of defence-signalling sectors grown in pot with calcined clay sterilized substrate) | 38-strain community identified as core microbiota after 16S rRNA sequencing | The authors established that immune signalling (mainly by salicylic acid) drives root colonization of the available soil microbial communities | Lebeis et al. (2015)^14^ |
| Tomato (potted experiments using sterilized soil under glasshouse conditions) | 4-strain community formed by rhizosphere isolated bacteria | The inoculated SynCom strongly activate host immune responses against *R. solanacearum* with greater extent than the individual strain. Dysbiosis of protective Gram-positive rhizosphere community can promote the disease incidence | Lee et al. (2021)^15^ |
| Wheat (potted experiments using autoclaved soil under glasshouse conditions) | 8-strain community formed by PGPR isolated from wheat rhizosphere | The inoculated SynCom increased wheat yield and biomass. Additionally, a soil-borne pathogen *Fusarium pseudograminearum* load in soil was significantly reduced by SynCom-mediated alteration of soil microbiome structure | Liu et al. (2022)^16^ |
| *A. thaliana* and Barley (plants grown in axenic conditions) | Two SynComs composed of two fungal models *Serendipita vermifera* and *Bipolaris sorokiniana* for both plants + 4-bacterial strain community in Arabidopsis and 26-bacterial strain community for Barley | Using SynCom with a fungal endophyte and core bacteria microbiota members for each plant, the authors demonstrated the hypothesis that the establishment of beneficial inter-kingdom interactions in the plant microbiota is an evolutionary and conserved trait leading to a synergistic protection against a soilborne fungal pathogen | Mahdi et al. (2022)^17^ |
| Alfalfa (plants grown in axenic conditions under different Nitrogen concentrations) | 10-strain community formed by Alfalfa leaves and flowers isolated bacteria | Researchers demonstrated that plants can shape the microbiome assembly depending on nitrogen concentration | Moccia et al. (2020)^18^ |
| Rice (seedlings grown in axenic conditions) | 10-strain community formed by root isolated PGPR bacteria | Inoculated seedlings showed higher growth rates than the uninoculated. Authors concluded that the use of simplified SynCom is useful to better understand microbe-microbe and microbe-plant synergistic interactions | Moronta-Barrios et al. (2018)^19^ |
| Maize (SynCom experments were performed on seedlings grown in axenic conditions) | 7-strain community representing three of the most dominant phyla found in maize roots | In this study the authors demonstrated the roles that each strain plays during the community assembly. Furthermore, they highlight the SynCom potential as useful approach to investigate how bacterial interspecies interactions affect both root microbiome assembly and beneficial hosts effects | Niu et al., (2017)^20^ |
| Sorghum (plants grown in axenic conditions) | Three SynComs by differently mixed a total of 53-strain community isolated from Brassicaceae roots (Finkel et al., 2020) | In this study, the SynCom approach allowed to identify that the *Variovorax* strains can protect sorghum growth from drought and from the activity of root growth inhibition bacterial strains. Additionally, data were compared with field experiments with some convergent results | Qi et al. (2022)^21^ |
| *A. thaliana* (plants grown in axenic conditions) | 185-strain community formed by bacteria isolated from Brassicaceae roots | In this study the effects of sublethal dose of Glyphosate (hormesis induction) on plant microbiome was observed using a SynCom approach. SynCom reduce hormesis effects due to some strains known as root growth inhibitors. In sum, glyphosate hormesis phenomenon is completely dependent by the microbiome composition | Ramirez-Villacis et al. (2020)^22^ |
| *Nicotiana attenuata* (grown in axenic conditions) | 5-strain community formed by native bacterial isolates isolated from the plant's natural habitat | The inoculated SynCom protects its host against fungal pathogens because of complementary traits of the five strains forming the multitaxa consortia | Santhanam et al. (2019)^23^ |
| Tomato (plants grown in pots with unsterilized substrate) | Two SynCom: 1) 15-strain community and 2) the simplified SynCom 1 to 5-strain community formed by root-derived bacteria isolated from the desert plant *Indigofera argentea* | The authors used non-sterile soil to mimic a realistic agricultural setting. Both SynComs successfully protected tomato plants against salt stress. This was coupled with a differential expression of salt stress-related genes and ion accumulation in inoculated tomato plants | Schmitz et al. (2022)^24^ |
| *A. thaliana* (plants grown in axenic conditions) and Tomato (plants grown in pots under controlled growth chamber) | Two SynComs formulated using 25-strain community each isolated from the rhizosphere of tomato plants grown in a suppressive compost | Both SynComs positively affected Tomato growth and suppressed *Fusarium* wilt symptoms. Conversely no or negative effects were observed for *Arabidopsis* *in vitro*. The authors concluded that the application of SynComs on poor substrates can yield reproducible plant phenotypes | Tsolakidou et al. (2019)^25^ |
| *A. thaliana* (mutants with absence of root-secreted phytoalexins, flavonoids and coumarins) | 22-strain community isolated from *Arabidopsis* roots | In this study, mechanisms of microbiome shaping by plant derived molecules were elucidated as mainly mediated by secreted coumarins | Voges et al. (2019)^26^ |
| Maize (grown *in vitro* and in gnotobiotic bags) | 7-strain community formed by maize root-colonizing bacteria | The authors demonstrated an ecological phenomenon where belowground microorganisms can influence the early growth of inbred and hybrid maize plants | Wagner et al. (2021)^27^ |
| Rice (*indica* and *japonica* varieties) | Two SynComs: 1) 16 bacteria from *indica*-enriched OTUs; 2) 3 bacteria from *japonica*-enriched OTUs | In this study the authors used SynComs to explain the historical observation of higher Nitrogen Use Efficiency (NUE) in *indica* varieties respect to *japonica* ones. The *indica* SynCom conferred higher growth effects respect the *japonica*-derived SynCom likely due to diverse bacteria-mediated transformation of organic nitrogen | Zhang et al. (2019)^28^ |
| Rape (potted experiments under controlled conditions) | 7-strain community formed by inorganic-phosphate-solubilizing bacteria (iPSBs) isolated from bulk soil | The SynCom was co-inoculated using biochar as carrier and mixed in substrate of potted rape. iPSB community was effective in rape growth features, P content and uptake | Zheng et al. (2019)^29^ |
| Radish (axenic seedlings) | 6-*Pseudomonas* strain community isolated from garlic rhizosphere in two growth periods (bolting and maturation) and different growth condition (diverse soil characteristics) | The authors used a top-down approach able to identify *Pseudomonas* as PGPR providing insights in how plants affect their microbial community assembly and how the microbiome influence growth and defence features, highlighting the SynCom exploitability for a sustainable agriculture | Zhuang et al. (2021)^30^ |
| Maize (grown in mesocosms with unsterilized soil under semi-controlled conditions) | 3-strain community formed by microbes isolated from tomato rhizosphere cultivated in arid and saline soil | SynCom increased tolerance to water stress improving ecophysiological parameters, biomass and yield production highlighting that SynCom selected for a particular stress could be suitable also for agronomical applications | Zoppellari et al. (2013)^31^ |

**Table S2.** Examples of beneficial soil microorganism applications to mitigate abiotic and biotic plant stresses during the last 15 years.

| **Beneficial**  **Soil-microorganism** | **Plant** | **Abiotic Stress** | **Biotic Stress** | **Reference** |
| --- | --- | --- | --- | --- |
| *Streptomyces rochei* IT20*S ;*  *Streptomyces vinaceusdrappus* SS14 | Pepper  (*Capsicum annuum* L.) |  | *Phytophthora capsici* | Abbasi et al.  (2020)^32^ |
| *Pseudomonas fluorescens* | Mung bean  (*Vigna radiata*) | Salinity |  | Ahmad et al.  (2013)^33^ |
| *Pseudomonas* sp. AKM-P6 | Sorghum  (*Sorghum vulgare*) | Heat |  | Ali et al.  (2009)^34^ |
| *Streptomyces* sp. | Wheat  (*Triticum aestivum*) | Salinity |  | Aly et al.  (2012)^35^ |
| *Pseudomonas* PS01 | Arabidopsis *(Arabidopsis thaliana)* | Salinity |  | Chu et al.  (2019)^36^ |
| *Arthrobacter arilaitensis;* *Streptomyces pseudovenezuelae* | Maize  (*Zea mais*) | Drought |  | Chukwuneme et al.  (2020)^37^ |
| *Bacillus amyloliquefaciens* B9601-Y2 | Maize  (*Z. mais*) |  | *Bipolaris maydis* | Cui et al.  (2019)^38^ |
| *Streptomyces albidoflavus*H12*;*  *Nocardiopsis aegyptica*H14 | Tomato  (*Solanum Lycopersicum* );  Carrot  (*Daucus Carota*) |  | *Fusarium oxysporum f. sp. adices-lycopersici;*  *Rhizoctonia solani* | Djebaili et al.  (2021)^39^ |
| *Streptomyces*sp*.* | Sugar beet  (*B. vulgaris*) |  | *Sclerotium rolfsii* | Errakhi et al.,  (2009)^40^ |
| *Bacillus*  *tequilensis* SSB07 | Soybean  (*Glycine max*) | Heat |  | Kang et al.  (2019)^41^ |
| *Bacillus cereus* | Tomato  (*S. lycopersicum*) |  | *Fusarium* o*xysporum;*  *Alternaria solani* | Karthika et al.  (2020)^42^ |
| *Bacillus* sp. BS061 | Cucumber  (*Cucumis sativus*)  Strawberry  (*Fragaria sp.*) |  | *Botrytis cinerea; Podosphaera xanthii* | Kim et al.  (2013)^43^ |
| *Streptomyces*sp*.* | Guava  (*Psidium guajava*) |  | *Fusarium oxysporum;*  *Alternaria solani.* | Mohandas et al.  (2013)^44^ |
| *Streptomyces* sp. PGPA39 | Tomato  (*S. lycopersicum*) | Salinity |  | Palaniyandi et al. (2014)^45^ |
| *Pseudomonas putida*PCI2 | Tomato  (*S. lycopersicum* ) |  | *Fusarium oxysporum* | Pastor et al.  (2016)^46^ |
| *Bradyrhizobium*  *Japonicum;*  *Bacillus thuringiensis* | Soybean  (*G. max*) | Drought |  | Prudent et al.  (2014)^47^ |
| *Streptomyces* sp. | Wheat  (*T. aestivum*) | Salinity |  | Sadeghi et al.  (2012)^48^ |
| *Amycolatopsis*sp*.* | Apple  *(Malus domestica)* |  | *Colletotrichum gloeosporioides* | Sadeghian et al.  (2016)^49^ |
| *Citricoccus zhacaiensis* B–4 | Onion  (*Allium cepa*) | Drought |  | Selvakumar et al. (2015)^50^ |
| *Streptomyces*sp*.* | Noce metella  (*Datura metel)* |  | *Tobacco Mosaic Virus* (TMV) | Sonya et al.,  (2012)^51^ |
| *Streptomyces rochei* SM3 | Chickpea  (*Cicer arietinum*) | Salinity |  | Srivastava et al. (2015)^52^ |
| *Streptomyces coelicolor*  DE07 ;  *Streptomyces*  *olivaceus* DE10 ;  *Streptomyces geysiriensis*  DE27 | Wheat  (*T. aestivum*) | Drought |  | Yandigeri et al. (2012)^53^ |

**References**

1 Armanhi JSL, de Souza RSC, Biazotti BB, Yassitepe JE de CT, Arruda P. Modulating drought stress response of maize by a synthetic bacterial community. *Frontiers in microbiology* 2021; : 3042.

2 Bai Y, Müller DB, Srinivas G *et al.* Functional overlap of the Arabidopsis leaf and root microbiota. *Nature* 2015; **528**: 364–369.

3 Bodenhausen N, Bortfeld-Miller M, Ackermann M, Vorholt JA. A synthetic community approach reveals plant genotypes affecting the phyllosphere microbiota. *PLoS genetics* 2014; **10**: e1004283.

4 Carlström CI, Field CM, Bortfeld-Miller M, Müller B, Sunagawa S, Vorholt JA. Synthetic microbiota reveal priority effects and keystone strains in the Arabidopsis phyllosphere. *Nature Ecology & Evolution* 2019; **3**: 1445–1454.

5 Castrillo G, Teixeira PJPL, Paredes SH *et al.* Root microbiota drive direct integration of phosphate stress and immunity. *Nature* 2017; **543**: 513–518.

6 Chai YN, Ge Y, Stoerger V, Schachtman DP. High‐resolution phenotyping of sorghum genotypic and phenotypic responses to low nitrogen and synthetic microbial communities. *Plant, Cell & Environment* 2021; **44**: 1611–1626.

7 Durán P, Thiergart T, Garrido-Oter R *et al.* Microbial interkingdom interactions in roots promote Arabidopsis survival. *Cell* 2018; **175**: 973–983.

8 Durán P, Reinstädler A, Rajakrut AL *et al.* A fungal powdery mildew pathogen induces extensive local and marginal systemic changes in the Arabidopsis thaliana microbiota. *Environmental Microbiology* 2021; **23**: 6292–6308.

9 Finkel OM, Salas-González I, Castrillo G *et al.* The effects of soil phosphorus content on plant microbiota are driven by the plant phosphate starvation response. *PLoS Biology* 2019; **17**: e3000534.

10 Finkel OM, Salas-González I, Castrillo G *et al.* A single bacterial genus maintains root growth in a complex microbiome. *Nature* 2020; **587**: 103–108.

11 Herrera Paredes S, Gao T, Law TF *et al.* Design of synthetic bacterial communities for predictable plant phenotypes. *PLoS biology* 2018; **16**: e2003962.

12 Hu J, Wei Z, Friman V-P *et al.* Probiotic diversity enhances rhizosphere microbiome function and plant disease suppression. *MBio* 2016; **7**: e01790-16.

13 Huang Y-H, Liu Y, Geng J *et al.* Maize root-associated niches determine the response variation in bacterial community assembly and function to phthalate pollution. *Journal of Hazardous Materials* 2022; : 128280.

14 Lebeis SL, Paredes SH, Lundberg DS *et al.* Salicylic acid modulates colonization of the root microbiome by specific bacterial taxa. *Science* 2015; **349**: 860–864.

15 Lee S-M, Kong HG, Song GC, Ryu C-M. Disruption of Firmicutes and Actinobacteria abundance in tomato rhizosphere causes the incidence of bacterial wilt disease. *The ISME journal* 2021; **15**: 330–347.

16 Liu H, Qiu Z, Ye J, Verma JP, Li J, Singh BK. Effective colonisation by a bacterial synthetic community promotes plant growth and alters soil microbial community. *Journal of Sustainable Agriculture and Environment* 2022; **1**: 30–42.

17 Mahdi LK, Miyauchi S, Uhlmann C *et al.* The fungal root endophyte Serendipita vermifera displays inter-kingdom synergistic beneficial effects with the microbiota in Arabidopsis thaliana and barley. *The ISME journal* 2022; **16**: 876–889.

18 Moccia K, Willems A, Papoulis S *et al.* Distinguishing nutrient‐dependent plant driven bacterial colonization patterns in alfalfa. *Environmental Microbiology Reports* 2020; **12**: 70–77.

19 Moronta-Barrios F, Gionechetti F, Pallavicini A, Marys E, Venturi V. Bacterial microbiota of rice roots: 16S-based taxonomic profiling of endophytic and rhizospheric diversity, endophytes isolation and simplified endophytic community. *Microorganisms* 2018; **6**: 14.

20 Niu B, Paulson JN, Zheng X, Kolter R. Simplified and representative bacterial community of maize roots. *Proceedings of the National Academy of Sciences* 2017; **114**: E2450–E2459.

21 Qi M, Berry JC, Veley KW *et al.* Identification of beneficial and detrimental bacteria impacting sorghum responses to drought using multi-scale and multi-system microbiome comparisons. *The ISME Journal* 2022; : 1–13.

22 Ramirez-Villacis DX, Finkel OM, Salas-González I *et al.* Root microbiome modulates plant growth promotion induced by low doses of glyphosate. *MSphere* 2020; **5**: e00484-20.

23 Santhanam R, Menezes RC, Grabe V, Li D, Baldwin IT, Groten K. A suite of complementary biocontrol traits allows a native consortium of root‐associated bacteria to protect their host plant from a fungal sudden‐wilt disease. *Molecular ecology* 2019; **28**: 1154–1169.

24 Schmitz L, Yan Z, Schneijderberg M *et al.* Synthetic bacterial community derived from a desert rhizosphere confers salt stress resilience to tomato in the presence of a soil microbiome. *The ISME Journal* 2022; : 1–14.

25 Tsolakidou M-D, Stringlis IA, Fanega-Sleziak N, Papageorgiou S, Tsalakou A, Pantelides IS. Rhizosphere-enriched microbes as a pool to design synthetic communities for reproducible beneficial outputs. *FEMS Microbiology Ecology* 2019; **95**: fiz138.

26 Voges MJ, Bai Y, Schulze-Lefert P, Sattely ES. Plant-derived coumarins shape the composition of an Arabidopsis synthetic root microbiome. *Proceedings of the National Academy of Sciences* 2019; **116**: 12558–12565.

27 Wagner MR, Tang C, Salvato F *et al.* Microbe-dependent heterosis in maize. *Proceedings of the National Academy of Sciences* 2021; **118**.

28 Zhang J, Liu Y-X, Zhang N *et al.* NRT1. 1B is associated with root microbiota composition and nitrogen use in field-grown rice. *Nature biotechnology* 2019; **37**: 676–684.

29 Zheng B-X, Ding K, Yang X-R *et al.* Straw biochar increases the abundance of inorganic phosphate solubilizing bacterial community for better rape (Brassica napus) growth and phosphate uptake. *Science of the total environment* 2019; **647**: 1113–1120.

30 Zhuang L, Li Y, Wang Z *et al.* Synthetic community with six Pseudomonas strains screened from garlic rhizosphere microbiome promotes plant growth. *Microbial biotechnology* 2021; **14**: 488–502.

31 Zoppellari F, Malusà E, Chitarra W, Lovisolo C, Spanna F, Bardi L. Improvement of drought tolerance in maize (Zea mays L.) by selected rhizospheric microorganisms. *Ital J Agrometeorol* 2014; **18**: 5–18.

32 Abbasi S, Safaie N, Sadeghi A, Shamsbakhsh M. Tissue-specific synergistic bio-priming of pepper by two Streptomyces species against Phytophthora capsici. *PloS one* 2020; **15**: e0230531.

33 Ahmad M, Zahir ZA, Nazli F, Akram F, Arshad M, Khalid M. Effectiveness of halo-tolerant, auxin producing Pseudomonas and Rhizobium strains to improve osmotic stress tolerance in mung bean (Vigna radiata L.). *Brazilian Journal of Microbiology* 2013; **44**: 1341–1348.

34 Ali SZ, Sandhya V, Grover M, Kishore N, Rao LV, Venkateswarlu B. Pseudomonas sp. strain AKM-P6 enhances tolerance of sorghum seedlings to elevated temperatures. *Biology and Fertility of Soils* 2009; **46**: 45–55.

35 Aly MM, El Sayed H, Jastaniah SD. Synergistic effect between Azotobacter vinelandii and Streptomyces sp. isolated from saline soil on seed germination and growth of wheat plant. *Journal of American Science* 2012; **8**: 667–676.

36 Chu TN, Tran BTH, Van Bui L, Hoang MTT. Plant growth-promoting rhizobacterium Pseudomonas PS01 induces salt tolerance in Arabidopsis thaliana. *BMC research notes* 2019; **12**: 1–7.

37 Chukwuneme CF, Babalola OO, Kutu FR, Ojuederie OB. Characterization of actinomycetes isolates for plant growth promoting traits and their effects on drought tolerance in maize. *Journal of Plant Interactions* 2020; **15**: 93–105.

38 Cui W, He P, Munir S *et al.* Efficacy of plant growth promoting bacteria Bacillus amyloliquefaciens B9601-Y2 for biocontrol of southern corn leaf blight. *Biological Control* 2019; **139**: 104080.

39 Djebaili R, Pellegrini M, Ercole C, Farda B, Kitouni M, Del Gallo M. Biocontrol of Soil-Borne Pathogens of Solanum lycopersicum L. and Daucus carota L. by Plant Growth-Promoting Actinomycetes: In Vitro and In Planta Antagonistic Activity. *Pathogens* 2021; **10**: 1305.

40 Errakhi R, Lebrihi A, Barakate M. In vitro and in vivo antagonism of actinomycetes isolated from Moroccan rhizospherical soils against Sclerotium rolfsii: a causal agent of root rot on sugar beet (Beta vulgaris L.). *Journal of Applied Microbiology* 2009; **107**: 672–681.

41 Kang S-M, Khan AL, Waqas M *et al.* Integrated phytohormone production by the plant growth-promoting rhizobacterium Bacillus tequilensis SSB07 induced thermotolerance in soybean. *Journal of Plant Interactions* 2019; **14**: 416–423.

42 Karthika S, Midhun SJ, Jisha M. A potential antifungal and growth-promoting bacterium Bacillus sp. KTMA4 from tomato rhizosphere. *Microbial Pathogenesis* 2020; **142**: 104049.

43 Kim Y-S, Song J-G, Lee I-K, Yeo W-H, Yun B-S. Bacillus sp. BS061 suppresses powdery mildew and gray mold. *Mycobiology* 2013; **41**: 108–111.

44 Mohandas S, Poovarasan S, Panneerselvam P *et al.* Guava (Psidium guajava L.) rhizosphere Glomus mosseae spores harbor actinomycetes with growth promoting and antifungal attributes. *Scientia Horticulturae* 2013; **150**: 371–376.

45 Palaniyandi S, Damodharan K, Yang S, Suh J. Streptomyces sp. strain PGPA39 alleviates salt stress and promotes growth of ‘Micro Tom’tomato plants. *Journal of applied microbiology* 2014; **117**: 766–773.

46 Pastor N, Masciarelli O, Fischer S, Luna V, Rovera M. Potential of Pseudomonas putida PCI2 for the protection of tomato plants against fungal pathogens. *Current microbiology* 2016; **73**: 346–353.

47 Prudent M, Salon C, Souleimanov A, Emery R, Smith DL. Soybean is less impacted by water stress using Bradyrhizobium japonicum and thuricin-17 from Bacillus thuringiensis. *Agronomy for sustainable development* 2015; **35**: 749–757.

48 Sadeghi A, Karimi E, Dahaji PA, Javid MG, Dalvand Y, Askari H. Plant growth promoting activity of an auxin and siderophore producing isolate of Streptomyces under saline soil conditions. *World Journal of Microbiology and Biotechnology* 2012; **28**: 1503–1509.

49 Sadeghian M, Bonjar GHS, Sirchi GRS. Post harvest biological control of apple bitter rot by soil-borne Actinomycetes and molecular identification of the active antagonist. *Postharvest Biology and Technology* 2016; **112**: 46–54.

50 Selvakumar G, Bhatt RM, Upreti KK, Bindu GH, Shweta K. Citricoccus zhacaiensis B-4 (MTCC 12119) a novel osmotolerant plant growth promoting actinobacterium enhances onion (Allium cepa L.) seed germination under osmotic stress conditions. *World Journal of Microbiology and Biotechnology* 2015; **31**: 833–839.

51 Mohamed Sonya H, Omran W, Shimaa MA-S, Al-Shehri A, Sadik A. Isolation and Identification of some halotolerant Actinomycetes having antagonistic activities against some plant pathogens (ie, Tobacco mosaic virus, Aspergillus Sp., Fusarium Sp.) from soil of Taif Governorate KSA. *Pakistan Journal of Biotechnology* 2012; **9**: 1–12.

52 Srivastava S, Patel JS, Singh HB, Sinha A, Sarma BK. Streptomyces rochei SM 3 induces stress tolerance in chickpea against Sclerotinia sclerotiorum and NaCl. *Journal of Phytopathology* 2015; **163**: 583–592.

53 Yandigeri MS, Meena KK, Singh D *et al.* Drought-tolerant endophytic actinobacteria promote growth of wheat (Triticum aestivum) under water stress conditions. *Plant Growth Regulation* 2012; **68**: 411–420.
